# Dietary Supplementation with *Mitragyna speciosa* Leaf Powder and *Saccharomyces cerevisiae* Improves Nutrient Digestibility, Rumen Fermentation Efficiency, and Microbial Nitrogen Supply in Thai Native Beef Cattle

**DOI:** 10.3390/ani16213488

**Published:** 2026-11-04

**Authors:** Sutthida Thiwato, Tanom Tathong, Anusorn Cherdthong, Kampanat Phesatcha

**Affiliations:** 1 Department of Animal Science, Faculty of Agriculture and Technology, Nakhon Phanom University, Nakhon Phanom 48000, Thailand; 2 Department of Food Technology, Faculty of Agriculture and Technology, Nakhon Phanom University, Nakhon Phanom 48000, Thailand; 3 Department of Animal Science, Faculty of Agriculture, Khon Kaen University, Khon Kaen 40002, Thailand

**Keywords:** kratom, phytogenic feed additive, direct-fed microbial, rumen fermentation, protozoa, microbial protein synthesis

## Abstract

This study evaluated the effects of *Mitragyna speciosa* leaf powder (MSP) and *Saccharomyces cerevisiae* (SC) supplementation on nutrient intake and digestibility, rumen fermentation, microbial populations, and microbial protein synthesis in Thai native beef cattle. Four female Thai native cattle (130.0 ± 7.4 kg body weight at the start of the experiment) were randomly assigned in a 4 × 4 Latin square design to four dietary treatments: (T1) total mixed ration (TMR) alone (control); (T2) TMR + MSP at 10 g/head/day; (T3) TMR + SC at 10 g/head/day; and (T4) TMR + MSP at 5 g/head/day + SC at 5 g/head/day. A TMR containing 14% crude protein was offered ad libitum throughout four 21-day periods. Total dry matter intake and nutrient intakes did not differ among treatments (*p* > 0.05), although the planned contrast between the control and the supplemented treatments indicated a small shortfall in the control for dry matter intake (*p* = 0.041). Apparent total-tract digestibility of dry matter, organic matter, crude protein, and acid detergent fiber was higher in supplemented animals than in the control (*p* < 0.05), and neutral detergent fiber digestibility tended to increase (*p* = 0.052). Ruminal pH increased, significantly so in the two treatments containing SC (*p* = 0.001), whereas ruminal ammonia nitrogen and blood urea nitrogen decreased with supplementation (*p* < 0.05). Total volatile fatty acid concentration was unchanged, but the propionate proportion increased and estimated methane production decreased (*p* < 0.05). Total bacterial counts increased and protozoal counts decreased with supplementation (*p* < 0.05), while fungal counts were unaffected. Urinary purine derivative excretion and absorption increased with supplementation, and microbial nitrogen supply rose from 24.66 to 34.21–35.93 g N/d (*p* = 0.002). The efficiency of microbial nitrogen supply rose from 15.44 to 19.30–20.07 g N/kg of organic matter digested in the rumen (*p* = 0.007). Supplementation with MSP and SC, alone or in combination at 5 g of each, therefore enhances nutrient digestibility and rumen fermentation efficiency in Thai native beef cattle without depressing intake.

## Simple Summary

Low-quality, fibrous feeds are used by smallholder farmers in tropical Asia, which reduces cattle’s ability to convert feed into meat and increases the amount of energy wasted as methane. An appealing substitute for antibiotic growth enhancers is natural feed additives. While brewer’s yeast (*Saccharomyces cerevisiae*) is frequently used to regulate rumen conditions, kratom (*Mitragyna speciosa*), a tree native to Southeast Asia, has leaves that are rich in alkaloids, phenolic compounds, and flavonoids. Four native Thai beef heifers were used in this investigation. They were either fed a total mixed feed or supplemented with yeast, kratom leaf powder, or both. Supplementation did not reduce the amount of feed the animals ate, and it improved the digestion of dry matter, protein, and fiber; raised the pH of the rumen in the treatments that contained yeast; increased the number of rumen bacteria while decreasing protozoa; shifted fermentation toward propionate; reduced estimated methane production; and increased the amount of microbial protein supplied to the animal. These findings show that yeast and kratom leaf powder, separately or in combination, can improve how well the ration is digested and how efficiently the rumen uses it. The study measured digestion and rumen function over four short periods, however, and did not measure liveweight gain, carcass quality or feed conversion, so better digestion should not be read as proof of better growth, nor is it yet known whether the alkaloids of kratom leave residues in meat or milk. Longer feeding studies that measure growth and residues are needed before either additive can be recommended for commercial use in tropical smallholder beef systems.

## 1. Introduction

In Southeast Asia, where smallholder systems predominate and cattle offer draught power, manure, savings, and meat, the raising of beef cattle supports rural livelihoods and food security [1]. Native breeds are highly valued in Thailand because of their ability to produce edible protein from crop leftovers and agro-industrial by-products such as rice straw and cassava processing wastes, as well as their resistance to heat and illness [2]. These same low-quality, fibrous feeds, however, are poorly broken down in the rumen, which limits the availability of microbial protein and diverts a significant amount of dietary energy to methane. Enhancing the efficacy of rumen fermentation is thus crucial for augmenting productivity in tropical beef systems. Due to the prohibition or stringent limitations on prophylactic and growth-enhancing antibiotics in numerous countries, research focus has transitioned to natural feed additives that can influence the rumen environment [3]. Two classifications have garnered notable attention: phytogenic additions originating from plant secondary compounds (PSCs) and direct-fed microbials (DFMs).

Many botanicals are rich in PSCs—alkaloids, flavonoids, polyphenols, condensed tannins, and saponins—that exert selective antimicrobial activity, particularly against rumen protozoa and methanogens [4]. Because these compounds interact with dietary fiber and protein and modify bacterial growth, they can shift fermentation pathways, reduce excessive ruminal proteolysis, and improve nitrogen retention [5], and they have been reported to improve nutrient digestibility, microbial protein synthesis, and antioxidant status in ruminants [6]. The evidence from tropical plant materials is consistent on this point. Dragon fruit (*Hylocereus undatus*) peel powder, which contains approximately 6–7% condensed tannins and 8–9% saponins, reduced ruminal protozoa by 66–70% and estimated methane production by 9–10% in Holstein crossbred bulls while increasing propionate and microbial protein synthesis [7]. Suppression of ruminal protozoa is one route by which such compounds act, and a meta-analysis of protozoal suppression in ruminants links it to a larger bacterial population and lower methane output [8]. Supplementation of beef cattle with tannin- and polyphenol-rich tropical leaves at only 10 g/day lowered protozoal counts and improved nitrogen retention without depressing intake or digestibility [9]. More broadly, saponin-containing plant materials have been shown to suppress ruminal protozoa, with associated changes in nitrogen metabolism and in methane production, although the size of the response varies with the saponin source and the dose supplied [10].

*Mitragyna speciosa* (Korth.) Havil., known in Thailand as kratom, is a native Southeast Asian tree of the Rubiaceae family that contains high concentrations of bioactive compounds. Ethanolic extracts of its leaves have been reported to contain 407.83 ± 2.50 mg gallic acid equivalents/g total phenolics, 194.00 ± 5.00 mg quercetin equivalents/g flavonoids, and 6.0–7.0% mitragynine [11]. Following its decriminalization in Thailand in 2021, kratom leaf has become widely and inexpensively available as an agricultural commodity, creating an opportunity for its use as an on-farm feed resource. Evidence in ruminants is now accumulating. In goats, dried kratom leaf supplementation linearly decreased the acetate proportion and estimated methane production while linearly increasing propionate and lowering ruminal ammonia nitrogen and blood urea nitrogen, indicating improved nitrogen utilization [12]; feeding kratom leaf powder at up to 6.66 g/day for 90 days produced no adverse effects on growth performance or hematology while increasing hot carcass weight, longissimus muscle area, and the monounsaturated fatty acid content of the meat [13]. In Thai indigenous beef cattle, the administration of kratom leaf pellets at a dosage of 10–30 g/head/day enhanced dry matter, organic matter, and fiber digestibility; elevated total volatile fatty acids and propionate levels; diminished ruminal ammonia nitrogen and projected methane emissions; augmented total bacterial counts and cellulolytic species while decreasing protozoa and methanogens; and improved the efficiency of microbial nitrogen supply [14]. In vitro, microencapsulated Mitragyna leaf extract enhanced dry matter degradability and propionate levels, diminished methane production, and altered the microbial community to favor cellulolytic bacteria while reducing *Methanobacteriales* [15].

DFMs, in contrast, act by supplying live microorganisms that stabilize the rumen environment. *S. cerevisiae* is the most widely used yeast DFM in ruminants; it scavenges oxygen, stimulates lactate-utilizing and cellulolytic bacteria, buffers ruminal pH, and thereby improves fiber degradation and microbial protein flow to the small intestine [3]. In Thai native beef cattle, active dry yeast at 1–3 g/head/day increased neutral and acid detergent fiber digestibility, raised total volatile fatty acids and propionate, reduced protozoa and methanogens, and increased *Fibrobacter succinogenes* and *Ruminococcus flavefaciens* [16].

Because the two additive classes act through distinct and potentially complementary mechanisms—PSCs suppressing protozoa and methanogens, yeast promoting cellulolytic and lactate-utilizing bacteria, and stabilizing ruminal pH—their combination may deliver equivalent or additive benefits at lower inclusion rates of each. To date, however, only one study has crossed a kratom-type phytogenic with a yeast DFM in ruminants: in goats, yeast at 0.5 g/kg DM and dried kratom leaf at 4.44 g/kg DM each improved nutrient digestibility, reduced protozoa and estimated methane, and increased propionate, with no interaction on intake or on bacterial and fungal populations [17]. Whether the same combination is effective in cattle—a host with a substantially larger rumen volume, longer retention time, and different microbial community—has not been examined, and the consequences for microbial protein synthesis in tropical native cattle have not been characterized. Therefore, the objective of the present study was to evaluate the effects of *M. speciosa* leaf powder and *S. cerevisiae*, supplemented alone or in combination, on feed intake, nutrient digestibility, rumen fermentation characteristics, rumen microbial populations, and microbial protein synthesis in Thai native beef cattle.

## 2. Materials and Methods

### 2.1. Ethical Approval

All animal procedures were reviewed and approved by the Institutional Animal Care and Use Committee of Nakhon Phanom University (approval number NPU003/2566, approved on 16 May 2023), in accordance with the Ethical Principles and Guidelines for the Use of Animals for Scientific Purposes, National Research Council of Thailand.

### 2.2. Preparation of Supplements

Fresh *M. speciosa* leaves were harvested, leaves were dried in a hot-air oven at 60 °C to constant weight, ground to pass a 1 mm screen, and stored in sealed opaque containers at ambient temperature until use to give the *M. speciosa* leaf powder (MSP) used in the experiment. The condensed tannin concentration of the MSP was determined by the acidified vanillin (vanillin–HCl) method [18] and expressed as catechin equivalents on a dry matter basis. The brewer’s yeast used as the SC supplement was *S. cerevisiae*, obtained from a local producer in Renu Nakhon District, Nakhon Phanom Province, Thailand, containing 2.9 × 10^9^ colony-forming units per gram. Because the yeast was sourced locally rather than from a commercial feed-additive supplier, no proprietary strain designation is available, and the strain was not characterized further in this study. Both supplements were weighed and offered on an air-dry basis.

### 2.3. Animals, Experimental Design, and Dietary Treatments

Four female Thai native beef cattle aged approximately 12 months and weighing 130.0 ± 7.4 kg at the start of the first period were randomly allocated to four dietary treatments in a 4 × 4 Latin square design. Treatments were T1, TMR alone (control); T2, TMR + MSP at 10 g/head/day; T3, TMR + SC at 10 g/head/day; and T4, TMR + MSP at 5 g/head/day + SC at 5 g/head/day. The 10 g/head/day inclusion was chosen as the lowest dose previously shown to be effective for *M. speciosa* leaf in this breed, where a leaf pellet fed at 10, 20 and 30 g/head/day to Thai native cattle of 128 ± 10 kg improved digestibility and fermentation at every level [14]; the same quantity, 10 g/head/day, has been used for tannin- and polyphenol-rich tropical leaves in beef cattle [9]. Keeping the total additive inclusion at 10 g/head/day in T2, T3 and T4 allowed the combination to be compared with each additive alone at an equal total inclusion rate rather than introducing a third dose level, and at approximately 0.2% of dry matter intake the quantity is one that a smallholder can weigh out by hand. Each supplement was hand-mixed into approximately 500 g of the morning total mixed ration and offered on its own; the remainder of the daily ration was offered only after this portion had been completely consumed, so that the full dose was ingested by every animal on every day of the experiment. A TMR formulated to contain 14.0% crude protein on a DM basis was prepared from rice straw, cassava top silage, and cassava peel silage (20% of DM each) together with cassava chip, soybean meal, leucaena leaf meal, rice bran, urea, molasses, a mineral mixture, salt, and sulfur (Table 1). The TMR was offered ad libitum twice daily at 07:00 and 16:00 h, with refusals allowed at approximately 10% of the amount offered. Clean drinking water and a mineral block were always available. Animals were housed in individual pens with free access to feed and water at the Department of Animal Science, Faculty of Agriculture and Technology, Nakhon Phanom University. The experiment was carried out between October 2023 and February 2024.

**Table 1:** Ingredient and chemical composition of the total mixed ration and chemical composition of *Mitragyna speciosa* leaves used in the experiment.

Item	Total Mixed Ration	*Mitragyna speciosa* Leaf Powder
Ingredient, % of DM		
Rice straw	20.0	–
Cassava top silage	20.0	–
Cassava peel silage	20.0	–
Cassava chip	16.0	–
Soybean meal	10.0	–
Leucaena leaf meal	7.0	–
Rice bran	4.0	–
Urea	1.6	–
Molasses	0.8	–
Mineral mixture ^1^	0.2	–
Salt	0.2	–
Sulfur	0.2	–
Total	100.0	–
Chemical composition		
Dry matter, % as fed	56.5	91.3
Organic matter, % of DM	88.7	93.2
Crude protein, % of DM	14.3	19.5
Ash, % of DM	11.3	6.8
Neutral detergent fiber, % of DM	58.6	51.4
Acid detergent fiber, % of DM	35.2	24.5
Condensed tannin, % of DM	–	15.3

DM = dry matter. ^1^ mineral and vitamin premix supplied per kg of premix: vitamin A, 10,000,000 IU; vitamin D, 1,600,000 IU; vitamin E, 70,000 IU; Fe, 50 g; Zn, 40 g; Mn, 40 g; Cu, 10 g; Co, 0.1 g; Se, 0.1 g. The dry matter content given for the total mixed ration is that of the representative pooled sample; the dry matter of the ration as offered varied between periods (57.5 to 60.5%, mean 58.5%), and dry matter intake in Table 2 was calculated against the value determined in each period, which is why the ratio of dry matter to fresh intake in Table 2 exceeds 56.5%.

**Table 2:** Effect of *Mitragyna speciosa* leaf powder and *Saccharomyces cerevisiae* supplementation on feed and nutrient intake in Thai native beef cattle.

Item	T1	T2	T3	T4	SEM	*p*-Value
Body weight, kg	141.00	141.00	140.25	139.75	0.83	0.669
Total intake, kg fresh weight/day	7.58	7.93	7.90	7.68	0.188	0.522
Total intake, kg DM/day	4.40	4.63	4.60	4.55	0.064	0.160
Total intake, % of body weight	3.12	3.31	3.30	3.26	0.052	0.123
Nutrient intake, kg/day						
Organic matter	3.93	4.11	4.09	4.07	0.086	0.506
Crude protein	0.62	0.65	0.65	0.64	0.009	0.124
Neutral detergent fiber	2.59	2.72	2.70	2.69	0.045	0.267
Acid detergent fiber	1.55	1.62	1.61	1.60	0.024	0.261

T1 = total mixed ration (TMR) without supplementation (control); T2 = TMR + *Mitragyna speciosa* leaf powder 10 g/head/day; T3 = TMR + *Saccharomyces cerevisiae* 10 g/head/day; T4 = TMR + *Mitragyna speciosa* leaf powder 5 g/head/day + *Saccharomyces cerevisiae* 5 g/head/day. SEM = standard error of the mean; DM = dry matter. Orthogonal contrast *p*-values (control vs. supplemented; MSP vs. SC; combination vs. the two single additives): body weight 0.512, 0.546, 0.422; total intake as fresh weight 0.278, 0.928, 0.341; dry matter intake 0.041, 0.792, 0.457; intake as % of body weight 0.028, 0.957, 0.539; organic matter 0.168, 0.844, 0.751; crude protein 0.027, 0.849, 0.742; neutral detergent fiber 0.070, 0.762, 0.727; acid detergent fiber 0.075, 0.782, 0.529.

The experiment comprised four periods of 21 days each, consisting of 14 days of dietary adaptation followed by 7 days of sample collection. Feed offered and refused was recorded daily throughout, and body weight was recorded at the beginning and end of each period. The dry matter content of the ration was determined on the feed actually offered in each period and rose from 57.5% in the first period to 60.5% in the fourth as the moisture content of the cassava top and peel silages fell, with a mean of 58.5% across the experiment; dry matter intake in Table 2 was therefore calculated period by period against those values, whereas the single figure of 56.5% given in Table 1 is the analysis of the representative pooled sample of the ration. Mean body weight rose from 130.0 ± 7.4 kg in the first period to 151.8 ± 7.9 kg in the fourth, with a mean of 140.5 kg across the experiment; individual animals gained between 20 and 25 kg over the 84 days of the trial. The period effect on body weight was highly significant (*p* < 0.001) whereas the treatment effect was not (*p* = 0.669), confirming that growth was balanced across treatments by the Latin square. Intake expressed as a percentage of body weight was calculated using the body weight of each animal recorded within each period.

### 2.4. Sample Collection and Chemical Analyses

Within the 7-day collection period, feces and urine were sampled; urine was collected as a single spot sample taken from each animal 4 h after the morning feeding on the last day of each period. Feces were obtained daily by grab sampling with rectal and vulval stimulation, pooled within animal and period on an equal wet-weight basis, dried at 60 °C for 72 h, and ground to pass a 1 mm screen.

Apparent total-tract nutrient digestibility was determined using acid-insoluble ash (AIA) as an internal marker according to Van Keulen and Young [19]. Duplicate subsamples of feed and feces were ashed, boiled in 2 N HCl for 5 min, filtered through ashless filter paper, and re-ashed at 550 °C to constant weight. Apparent digestibility of nutrient *i* was calculated as: digestibility (%) = 100 − [100 × (AIA in feed/AIA in feces) × (nutrient *i* in feces/nutrient *i* in feed)].

Feed, refusal, and fecal samples were analyzed for dry matter (DM), ash, and crude protein (CP) according to AOAC [20]. Organic matter (OM) was calculated as 100 − ash. Neutral detergent fiber (NDF) and acid detergent fiber (ADF) were determined according to Goering and Van Soest [21] with the modifications described by Van Soest et al. [22]; NDF was assayed without heat-stable α-amylase and is expressed inclusive of residual ash.

On the last day of each period, rumen fluid was collected 4 h after the morning feeding by a stomach tube connected to a vacuum pump, with the first 100 mL discarded to avoid saliva contamination. Ruminal pH and temperature were measured immediately using a portable pH and temperature meter (HI 8424 microcomputer, Hanna Instruments Pte. Ltd., 161 Kallang Way, Singapore). Each sample was filtered through four layers of cheesecloth and divided into subsamples. One subsample was preserved with 1 M H_2_SO_4_ for determination of volatile fatty acids (VFA) by high-performance liquid chromatography according to Samuel et al. [23] and of ammonia nitrogen (NH_3_-N) by the micro-Kjeldahl distillation procedure of AOAC [20]. A second subsample was fixed in 10% formalin for enumeration of total bacteria, protozoa, and fungal zoospores by the direct count method using a Bürker counting chamber under a light microscope.

Blood was collected from the jugular vein 4 h after the morning feeding into tubes containing EDTA, centrifuged at 3000× *g* for 10 min at 4 °C, and the plasma stored at −20 °C until analysis of blood urea nitrogen (BUN) according to Crocker [24].

### 2.5. Estimation of Methane Production and Microbial Protein Synthesis

Ruminal methane production was estimated stoichiometrically from the molar VFA profile according to Moss et al. [25]:

CH_4_ = 0.45 (acetate) − 0.275 (propionate) + 0.40 (butyrate)

One spot urine sample was collected from each animal in each period, at the same time as rumen sampling and therefore 4 h after the morning feeding, immediately acidified to pH < 3 with 5 M H_2_SO_4_, and stored at −20 °C until analysis. Microbial protein supply was estimated from the urinary excretion of purine derivatives following the technique of Chen and Gomes [26]. Allantoin and uric acid were both determined, and total purine derivative excretion was taken as their sum; xanthine and hypoxanthine were not quantified, because they occur only in trace quantities in cattle urine and the FAO/IAEA laboratory manual for this method specifies the analysis of allantoin and uric acid alone for this species [27]. Creatinine was determined on the same samples. Daily urine volume was estimated from the creatinine concentration of the spot urine samples [28,29] as urine volume (L/d) = creatinine excretion coefficient (mg/kg BW/d) × body weight (kg)/urinary creatinine concentration (mg/L), with a creatinine excretion coefficient of 29 mg/kg BW/d. That value was adopted because the animals were growing heifers offered a roughage-based ration, a category for which creatinine excretion has been reported at 29.7 and 30.0 mg/kg BW/d on forage diets and at 28.6 mg/kg BW/d as the mean across heifers and cows [30]. Urinary creatinine concentration ranged from 31.8 to 42.5 mg/dL across animals and periods, and the estimated daily urine volume from 9.42 to 12.95 L/d, equivalent to 68 to 91 mL/kg body weight per day. The amount of purine derivatives absorbed (X, mmol/d) was calculated from the amount excreted in urine (Y, mmol/d) using the relationship of Chen and Gomes [26], and microbial nitrogen supply (MN, g N/d) was derived from absorbed purine derivatives as MN = 70X/(0.83 × 0.116 × 1000) [26,31]. The efficiency of microbial nitrogen supply (EMNS, g N/kg of OM apparently digested in the rumen, OMDR) was calculated as MN (g N/d)/DOMR (kg/d), where DOMR was computed for each animal within each period as organic matter intake × total-tract organic matter digestibility × 0.65. The factor of 0.65 is the one specified for this calculation in the FAO/IAEA laboratory manual [27] and derives from the Agricultural Research Council feeding standards for ruminant livestock [32].

### 2.6. Statistical Analysis

All data were analyzed as a 4 × 4 Latin square design using the GLM procedure of SAS 9.4 (SAS Institute Inc., Cary, NC, USA) [33] according to the model:

Y*ijk* = μ + M*i* + A*j* + P*k* + ε*ijk*

where Y*ijk* is the observation from animal *j* receiving diet *i* in period *k*; μ is the overall mean; M*i* is the effect of treatment (*i* = 1, 2, 3, 4); A*j* is the effect of animal (*j* = 1, 2, 3, 4); P*k* is the effect of period (*k* = 1, 2, 3, 4); and ε*ijk* is the residual error. Rumen fluid, blood and urine were each sampled once per animal and period, 4 h after the morning feeding, so that single observation is the one entering the model above; sampling time was therefore not a factor in the analysis and no time by treatment interaction could be tested. Treatment means were separated using Tukey’s honestly significant difference test. The standard error of a treatment mean given in every table is the square root of the residual mean square of that same analysis divided by the four observations contributing to each treatment mean, so that the standard errors and the probabilities reported in a table derive from one analysis. Three pre-planned orthogonal contrasts were also tested: the control versus the mean of the three supplemented treatments; MSP alone versus SC alone; and the combination versus the mean of the two single additives. Residuals were examined for normality with the Shapiro–Wilk test and for homogeneity of variance by inspection of residual plots. Differences were declared significant at *p* < 0.05, and 0.05 ≤ *p* < 0.10 was considered a tendency. With four animals and six residual degrees of freedom, the design has limited power to detect moderate treatment differences; this constraint is considered further in the Discussion.

## 3. Results

### 3.1. Chemical Composition of the Diets

The ingredient and chemical composition of the TMR and the chemical composition of the MSP are presented in Table 1. The TMR was formulated from 60% roughage (rice straw, cassava top silage, and cassava peel silage, 20% of DM each) and 40% concentrate ingredients and contained 56.5% DM and, on a DM basis, 88.7% OM, 14.3% CP, 11.3% ash, 58.6% NDF, and 35.2% ADF. The MSP contained 91.3% DM, and on a DM basis, 93.2% OM, 19.5% CP, 6.8% ash, 51.4% NDF, and 24.5% ADF, and its condensed tannin concentration was 15.3% of DM, indicating that kratom leaf powder is a moderately high-protein, high-fiber plant material rich in condensed tannins.

### 3.2. Feed and Nutrient Intake

Neither total intake expressed as fresh weight, dry matter, nor percentage of body weight differed among treatments (*p* > 0.05; Table 2). Dry matter intake averaged 4.54 kg/day and, calculated within each animal and period, 3.25% of body weight; mean body weight across the experiment was 140.5 kg and did not differ among treatments (*p* = 0.669). Intakes of organic matter, crude protein, neutral detergent fiber, and acid detergent fiber were likewise not separated by Tukey’s test (*p* > 0.05). The planned contrast between the control and the mean of the three supplemented treatments was nevertheless significant for dry matter intake (*p* = 0.041), intake as a percentage of body weight (*p* = 0.028) and crude protein intake (*p* = 0.027), and approached significance for neutral and acid detergent fiber intake (*p* = 0.070 and *p* = 0.075); this reflects a small and consistent numerical shortfall of 0.15 to 0.23 kg DM/day in the control rather than any depression in the supplemented animals. Neither MSP nor SC at the levels used therefore exerted any palatability or intake-depressing effect.

### 3.3. Nutrient Digestibility

Apparent total-tract digestibility was consistently improved by supplementation (Table 3). Relative to the control, DM digestibility increased from 60.40% to 64.77–65.40% (*p* = 0.040), OM digestibility from 62.60% to 65.40–67.40% (*p* = 0.034), CP digestibility from 56.85% to 63.77–65.00% (*p* < 0.001), and ADF digestibility from 31.57% to 40.25–41.12% (*p* = 0.002). NDF digestibility increased from 55.48% to 59.17–60.13%, which approached but did not reach the conventional threshold of significance (*p* = 0.052). The contrast between the control and the mean of the three supplemented treatments was significant for every fraction, neutral detergent fiber included (*p* ≤ 0.011). By Tukey’s test the control was separated from all three supplemented treatments for CP and ADF digestibility, whereas for DM and OM digestibility only one supplemented treatment was separated from the control and the other two took intermediate positions, which is the expected consequence of six residual degrees of freedom. Tukey’s test detected no differences among the three supplemented treatments for any digestibility measure; the only contrast to separate them was that between the combination and the mean of the two single additives for crude protein digestibility, where the combination was marginally the lower (63.77% against a mean of 64.85% for the two single additives; *p* = 0.049).

### 3.4. Rumen Fermentation Characteristics and Blood Urea Nitrogen

Ruminal pH differed among treatments (*p* = 0.001; Table 4), rising from 6.48 in the control to 6.60 with MSP alone, which Tukey’s test did not separate from the control, and to 6.80–6.83 in the two treatments containing yeast; the MSP versus SC contrast was correspondingly significant (*p* = 0.007), the only variable in the study for which the two additives differed significantly from one another; total bacterial counts showed a tendency in the same direction (*p* = 0.069). Ruminal ammonia nitrogen decreased from 16.65 mg/dL in the control to 12.08–13.10 mg/dL in supplemented animals (*p* < 0.001), and blood urea nitrogen followed the same pattern, declining from 14.50 mg/dL in the control to 11.75 and 12.25 mg/dL in T2 and T4 respectively, with T3 taking an intermediate value of 12.90 mg/dL (*p* = 0.023). Ruminal temperature was monitored at every sampling to confirm that the samples were representative and were not affected by treatment; it is not tabulated.

Total VFA concentration was similar across treatments (102.33–103.15 mmol/L; *p* = 0.957; Table 5). The proportion of acetate tended to decline with supplementation (66.40 in the control to 64.40–65.65 mol/100 mol; *p* = 0.097), whereas propionate increased from 19.88 to 21.93–22.28 mol/100 mol (*p* = 0.001). Butyrate was unaffected (*p* = 0.180). The acetate to propionate ratio therefore fell from 3.34 in the control to 2.94–2.96 in supplemented animals (*p* < 0.001), and estimated methane production decreased from 29.90 to 28.25–28.42 mol/100 mol (*p* = 0.001).

### 3.5. Rumen Microbial Populations

Total bacterial counts were markedly increased by supplementation, from 2.40 × 10^9^ cells/mL in the control to 3.60–4.45 × 10^9^ cells/mL (*p* = 0.009; Table 6); by Tukey’s test the rise was significant in T2 and T4, with T3 taking an intermediate position. Conversely, protozoal counts were reduced from 5.60 × 10^6^ to 2.20–2.70 × 10^6^ cells/mL (*p* = 0.002). Fungal zoospore counts did not differ among treatments (*p* = 0.790).

### 3.6. Microbial Protein Synthesis

Urinary purine derivative excretion and microbial nitrogen supply were both increased by supplementation (Table 7). Total purine derivative excretion rose from 44.58 mmol/d in the control to 55.63–57.69 mmol/d (*p* = 0.002), and the quantity absorbed increased correspondingly from 33.92 to 47.05–49.43 mmol/d (*p* = 0.002). Microbial nitrogen supply increased from 24.66 g N/d in the control to 34.21–35.93 g N/d, a gain of 38.7–45.7% (*p* = 0.002), equivalent to a microbial crude protein supply of 154.1 g/d in the control against 213.8–224.6 g/d in supplemented animals. Organic matter apparently digested in the rumen also increased, from 1.60 to 1.73–1.79 kg/d (*p* = 0.008). The efficiency of microbial nitrogen supply was 15.44 g N/kg OMDR in the control against 19.30–20.07 g N/kg OMDR in supplemented animals, an improvement of 25.0–30.0% (*p* = 0.007). The same pattern held for every variable in Table 7: the control versus supplemented contrast was significant throughout (*p* ≤ 0.002), while neither the contrast between MSP and SC alone (*p* ≥ 0.40) nor that between the combination and the mean of the two single additives (*p* ≥ 0.13) approached significance, so the difference lies between the control and supplementation rather than among the supplemented treatments. The underlying assay values were consistent with the method: allantoin accounted for 81.6–85.2% of total purine derivatives (*p* = 0.424), within the 80–85% proportion expected in cattle, and uric acid excretion did not differ among treatments (8.18–9.00 mmol/d, *p* = 0.866), so the treatment response resided in allantoin (36.40 against 46.95–49.04 mmol/d, *p* = 0.003).

## 4. Discussion

The absence of any treatment effect on feed intake is an important practical finding. Plant secondary compounds, particularly condensed tannins and saponins, are frequently associated with reduced palatability and depressed intake when supplied at high concentrations. In the present study, MSP was supplied at only 10 g/head/day—approximately 0.2% of dry matter intake—which was evidently well below any threshold for astringency or feed aversion. The same conclusion has been drawn for other tannin-rich tropical leaves fed to beef cattle at 10 g/day, which altered rumen fermentation without changing intake or digestibility [9], and for kratom leaf pellet fed to Thai native beef cattle at up to 30 g/head/day [14]. The benefits observed here were therefore achieved through modification of rumen microbial function rather than through any nutrient contribution or intake response.

The concurrent improvement in DM, OM, CP and fiber digestibility, achieved at constant intake, points to enhanced microbial degradative capacity in the rumen. Two mechanisms are consistent with the microbial data. First, the 1.50- to 1.85-fold increase in total bacterial counts, coupled with the 52–61% reduction in protozoal numbers, indicates a partial defaunation effect. Rumen protozoa engulf and digest bacteria, and their suppression is generally accompanied by an increase in bacterial biomass; a meta-analysis of defaunation studies reported a 6% rise in ruminal bacterial concentration [8]. Because bacteria were enumerated here by direct microscopy, which counts cells without distinguishing functional groups, these data are consistent with, but do not demonstrate, a shift in fibrolytic populations. The condensed tannins quantified in the kratom leaf powder offer a partial explanation for the efficacy of treatments containing MSP; saponins were not measured in the batch fed here, so any contribution from that class is inferred from published values for the same material, 8.2 to 11.2% of DM in a leaf pellet fed to this breed [14] and 5.21% in dried leaf fed to goats [17], rather than demonstrated in the present study. Comparable protozoal inhibition has been documented with dragon fruit peel powder in Holstein crossbred bulls [7] and with tannin- and polyphenol-laden foliage in beef cattle [9], and the same meta-analysis found that phytochemical treatments raised propionate by 5.4% and lowered methane by 17.9% [8]. The magnitude of the responses obtained here closely parallels that reported for kratom leaf pellet in the same breed [14], where nutrient digestibility, propionate and cellulolytic bacteria increased and protozoa, ammonia nitrogen and estimated methane decreased. Second, yeast supplementation stimulates cellulolytic and lactate-utilizing bacteria and scavenges oxygen at the feed particle surface, creating conditions more favorable for bacterial attachment and fiber degradation [3,16]. It is notable that active dry yeast alone in Thai native beef cattle improved only fiber digestibility, leaving DM, OM and CP digestibility unchanged [16], whereas in the present study all four fractions improved, consistent with the two additive classes acting on complementary components of the microbial community. That T2, T3 and T4 could not be separated statistically from one another for any digestibility, fermentation or microbial protein measure by Tukey’s test—ruminal pH was the single exception, being higher in the two yeast-containing treatments than with MSP alone—is consistent with the two additives, although mechanistically distinct, acting on a shared limiting step; this cannot establish equivalence, however, because the power to separate the supplemented treatments was low and the design contained no single-additive arms at 5 g against which the combination could be judged. It should be noted that the basal TMR itself contained leucaena leaf meal, a tannin-containing tropical legume, so all treatments including the control received a background load of plant secondary compounds; the responses reported here therefore represent the incremental effect of MSP and SC over an already partially tannin-supplemented diet, which may explain why the magnitude of the protozoal and methane responses was moderate.

The protozoal response deserves separate comment, because it was not confined to the treatments containing kratom. Protozoal numbers fell to a similar extent in T3, which supplied *S. cerevisiae* alone and contained no supplementary plant secondary compounds, as in T2 and T4. *S. cerevisiae* is not known to be directly protozoacidal, so the decline in T3 must be indirect, and attributing protozoal suppression chiefly to kratom-derived phytochemicals would over-read the data. Two indirect routes are plausible in this particular ration. The diet supplied 16% cassava chip, and ruminal protozoa, *Entodinium* species in particular, engulf starch granules and depend heavily on a rapidly fermentable starch supply; live yeast competes for soluble sugars and stimulates lactate-utilizing bacteria, which blunts the post-prandial starch and lactate surge and therefore narrows the substrate niche on which protozoa in a cassava-based diet depend. In addition, yeast scavenges oxygen at the feed particle surface and lowers redox potential, conditions that favor strict anaerobic bacteria and alter the competitive balance between bacteria and protozoa. The most defensible reading of the present data is therefore that the two additives reduced protozoal numbers by different routes that converge on a similar outcome in this diet, rather than that one mechanism explains all three supplemented treatments.

The condensed tannin concentration of the MSP, 15.3% of DM, is the value measured on the batch actually fed, by the acidified vanillin method against catechin [18]. It sits at the upper end of the published range for this material: 12.1 to 13.4% of DM has been reported for an *M. speciosa* leaf pellet fed to the same breed [14] and 8.28% for dried kratom leaf fed to goats [17]. Two factors account for the spread. The vanillin reaction against a catechin standard responds to flavan-3-ol monomers as well as to polymers, and kratom leaf is exceptionally rich in that class, carrying 194 mg quercetin equivalents of flavonoids and 408 mg gallic acid equivalents of total phenolics per gram [11], so any flavan-3-ol-reactive assay reads high in this material relative to a proanthocyanidin-specific method. Leaf maturity, harvest season and provenance also move plant secondary compound concentrations by two- to threefold in tropical foliage. Whichever figure is taken, the concentration deserves scrutiny in relation to the dose fed. At 10 g/head/day, MSP supplied only 1.53 g of condensed tannin per animal per day, equivalent to 0.033% of dry matter intake, and the combination treatment supplied half that amount. This is one to two orders of magnitude below the 1–4% of dietary DM at which condensed tannins are conventionally held to suppress protozoa and shift fermentation, and the basal diet itself contained leucaena leaf meal at 7% of DM, approximately 320 g/head/day, in every treatment, including the control. The condensed tannin dose alone, therefore, cannot account for the responses observed, and this conclusion does not depend on which condensed tannin concentration is accepted: at the 8.28% reported elsewhere [17] the dose would have supplied 0.83 g/d, or 0.018% of dry matter intake, which is further below the active range still. Two considerations resolve this apparent paradox. First, kratom leaf is distinguished less by its tannin content than by its indole alkaloids, chiefly mitragynine and 7-hydroxymitragynine, together with a high flavonoid and total phenolic concentration [11], and these compounds are pharmacologically active at far lower doses than tannins; a low-dose alkaloid-mediated effect would be consistent with the finding that microencapsulated *Mitragyna* leaf extract altered fermentation and reduced *Methanobacteriales* in vitro at very small inclusion rates [15]. Neither the alkaloid nor the total phenolic concentration of the batch of leaf powder fed here was determined, however, and the values quoted above are drawn from the literature on the species; this explanation is therefore advanced as a hypothesis, to be tested by quantifying mitragynine and total phenolics in the material fed and relating them to the response measured, and not as a mechanism demonstrated by the present data. Second, and more decisively, T3 contained no plant secondary compounds at all beyond the leucaena background, yet produced digestibility, protozoal and microbial protein responses indistinguishable from the MSP treatments, and a higher ruminal pH than MSP alone. The magnitude of the response was therefore not governed by plant secondary compound dose, which argues that both additives converged on the same limiting step in this diet—most plausibly the ruminal pH and nitrogen-synchrony constraint imposed by the cassava- and urea-based basal ration—rather than that either acted through a dose-dependent antimicrobial mechanism. This interpretation is testable, and a dose–response study of MSP spanning 10 to 40 g/head/day would establish whether the response observed here is at, below, or above the plateau.

Ruminal pH rose from 6.48 in the control to 6.60–6.83 with supplementation, significantly so only in the two treatments that contained yeast: MSP alone raised the mean by 0.13 units, which Tukey’s test did not separate from the control. The control animals were not acidotic: at 6.48 the mean already lay above the pH of approximately 6.2 below which fibrolytic bacteria such as *Fibrobacter succinogenes* and *Ruminococcus* spp. are inhibited. The change is therefore better described as a shift further into the range that favors cellulolysis, generally placed above pH 6.4, than as the correction of an existing constraint. The higher pH observed in the yeast-containing treatments, and the absence of a significant rise with MSP alone, is consistent with the well-documented capacity of *S. cerevisiae* to compete with lactate-producing bacteria for soluble sugars and to stimulate lactate utilization by *Megasphaera elsdenii* and *Selenomonas ruminantium* [3]. The pH response therefore offers a plausible explanation for the parallel improvement in fiber digestibility observed in the present study, which reached significance for ADF and remained a tendency for NDF.

The composition of the basal diet makes this pH response particularly informative. The TMR contained 16% cassava chip, a highly fermentable starch source, together with 1.6% urea and 0.8% molasses, so the control animals were exposed to a rapid post-prandial release of both glucose and ammonia. This is consistent with the control having simultaneously the lowest ruminal pH (6.48) and the highest NH3-N (16.65 mg/dL) and BUN (14.50 mg/dL) of the four treatments: rapid starch fermentation generates lactate and volatile fatty acids faster than they are absorbed or buffered, while urea hydrolysis by bacterial urease releases ammonia far faster than the microbial population on a fibrous substrate can assimilate it, so the surplus is absorbed across the rumen wall and converted to urea in the liver. Cassava-based tropical rations are well documented to produce this coupling of ruminal pH and nitrogen dynamics: in crossbred Holstein cattle offered cassava-based diets, ruminal pH remained between 6.54 and 6.76 while ruminal ammonia concentration varied with the cassava source fed [34], and urea- and molasses-treated cassava pulp has been reported to alter rumen fermentation and raise microbial protein synthesis in beef cattle offered cassava-based rations [35]. Against this background, the additive-driven rise in pH to 6.80–6.83 in the yeast-containing treatments is consistent with a reduced post-prandial acid load and may contribute to the improvement in fiber digestibility. This reading should be treated with caution, because ruminal pH was recorded at a single time point, 4 h after feeding, which cannot describe the post-prandial pH profile or the time spent below any given threshold. The simultaneous fall in NH3-N is best read as better synchrony between the rapid nitrogen release from urea and the microbial capacity to capture it, since a larger bacterial population with more digested organic matter available provides a larger sink for ammonia. Substituting cassava for rice straw in a total mixed ration silage has likewise been shown to raise total volatile fatty acids and the propionate to acetate ratio while linearly reducing enteric methane in Holstein crossbred bulls [36].

The concurrent reduction in ruminal ammonia nitrogen and blood urea nitrogen indicates more efficient ruminal nitrogen capture. Protozoa are major agents of intraruminal protein turnover through bacterial predation, and their suppression reduces the recycling of microbial nitrogen to ammonia [5]. The same coupled reduction in NH3-N and BUN was reported in goats supplemented with dried kratom leaf [12] and in beef cattle given tannin- and polyphenol-rich leaves, where it was accompanied by increased nitrogen retention and reduced total nitrogen excretion [9]. In addition, condensed tannins form pH-reversible complexes with dietary protein that resist ruminal degradation but dissociate in the abomasum, shifting protein digestion to the post-ruminal compartment. At the condensed tannin intake supplied here, however, this mechanism can have made only a marginal contribution, for the reasons set out above, and it cannot account for the response in T3, which received no supplementary tannin at all. Importantly, ammonia concentrations in all treatments (12.08–16.65 mg/dL) remained above the 8–10 mg/dL threshold generally regarded as necessary to sustain maximal microbial growth on tropical roughage-based diets, so the reduction observed here reflects improved nitrogen utilization rather than a nitrogen deficiency. The parallel decline in BUN, which reflects hepatic ureagenesis from absorbed ammonia, corroborates this interpretation and implies reduced urinary nitrogen loss—an outcome of both economic and environmental value.

The shift in the VFA profile, in which total VFA concentration was unchanged but propionate increased at the expense of acetate, is the classic signature of protozoal suppression and is characteristic of a more energetically efficient fermentation. Propionate is the principal glucogenic precursor in ruminants and is also a competing hydrogen sink; its increase necessarily reduces the metabolic hydrogen available for methanogenesis. The corresponding 4.9–5.5% reduction in estimated methane production is directionally consistent with, although smaller in magnitude than, the responses reported for dried kratom leaf in goats [12], for kratom leaf pellet in Thai native beef cattle [14], and for microencapsulated *Mitragyna* leaf extract in vitro, where methane fell at every incubation time point alongside a reduction in *Methanobacteriales* [15]. The reduction in protozoal numbers provides a second, independent route to lower methanogenesis, since methanogens associated ecto- and endosymbiotically with protozoa are estimated to account for 9–25% of ruminal methane production; consistent with this, a meta-analysis of defaunation strategies reported an 18.3% reduction in methane with complete defaunation [8]. It must be emphasized, however, that methane in the present study was estimated stoichiometrically from the VFA profile [25] and was not measured directly; whole-animal measurements of tannin- and saponin-based additives in tropical finishing cattle have shown reductions of the order of 15–17% [37], so estimated and measured responses need not coincide. The magnitude of the reduction reported here should therefore be interpreted as indicative rather than quantitative, and confirmation by respiration chamber, sulfur hexafluoride tracer or in vitro gas production techniques is warranted.

The microbial protein data reinforce this picture. Urinary purine derivative excretion, purine derivative absorption and the derived microbial nitrogen supply all increased with supplementation, which is internally consistent with the increased bacterial counts, the higher ruminal pH and the improved organic matter digestibility: a larger and more active bacterial population digesting more organic matter delivers more microbial protein to the small intestine. The gain in microbial nitrogen supply, from 24.66 to 34.21–35.93 g N/d, corresponds to a microbial crude protein supply of about 154 g/d in the control against 214–225 g/d in supplemented animals, a substantial addition of metabolizable protein for an animal consuming 0.62–0.65 kg of crude protein daily. Because nitrogen intake differed only marginally between treatments (0.62 versus 0.64–0.65 kg crude protein per day, a difference of 4–5% that reached significance only in the planned contrast and not in the overall test), this represents a real gain in the efficiency with which dietary nitrogen was converted into microbial protein rather than a consequence of feeding more protein; the increase also coincided with lower ruminal ammonia nitrogen and lower blood urea nitrogen, which is the pattern expected when ammonia is captured into microbial cells rather than absorbed and excreted as urea. Two assumptions temper these estimates, and both introduce systematic rather than random uncertainty. First, daily urine volume was estimated from creatinine concentration in spot samples rather than by total collection, so any departure of the assumed creatinine excretion rate from the true rate scales every purine derivative endpoint in the same direction. The measured urinary creatinine concentrations, 31.8 to 42.5 mg/dL, describe dilute urine, and the volumes they imply, 9.42 to 12.95 L/d or 68 to 91 mL/kg body weight per day, lie at the high end of what would be expected for cattle of this size, and three features of the protocol push the estimate in that direction: the ration was offered at 56.5% dry matter, so a substantial water load accompanied the feed; the diet carried 1.6% urea, with free access to water and to a mineral block, which imposes an osmotic load on the kidney; and the spot sample was taken 4 h after feeding, at the post-prandial peak of water turnover, when urine is most dilute and a creatinine-based estimate of volume is correspondingly at its highest. The absolute purine derivative excretion reported here should therefore be read as an upper estimate. Two observations limit the consequence. The derived quantities nevertheless fall within the conventional physiological range, a microbial crude protein supply of 154 to 225 g/d and an efficiency of 15.4 to 20.1 g N/kg OMDR against the 15 to 30 g N/kg OMDR usually reported for cattle; and although the endogenous term of the Chen and Gomes equation, 0.385 × BW^0.75^, is a constant subtraction rather than a multiplier, so that an error in the assumed coefficient does not displace the four treatment means in strict proportion, the sensitivity analysis set out below shows that the displacement is of similar relative magnitude in every treatment and leaves their ranking and separation unchanged. Varying the coefficient by ±20% around the 29 mg/kg BW/d adopted here changes the absolute microbial nitrogen supply by 27 to 31% in either direction—the spread across treatments, rather than a single proportional factor, arising from the constant endogenous term—but leaves the ranking of the treatments, the separation of the control from the supplemented treatments and the probabilities unaffected (*p* = 0.002 for microbial nitrogen supply and *p* ≤ 0.008 for its efficiency at every value tested), and the relative improvement over the control remains between 35 and 53%. Second, the efficiency term depends on the assumption that 65% of total-tract organic matter digestion occurs in the rumen; the denominator was nevertheless computed for each animal within each period from its own organic matter intake and total-tract organic matter digestibility, so the efficiency values carry the same animal-level variation as the numerator. A sensitivity analysis across the range 0.55 to 0.75 gives efficiencies of 18.24, 22.81, 23.72 and 23.53 g N/kg OMDR at 0.55 and 13.38, 16.73, 17.39 and 17.26 g N/kg OMDR at 0.75 for T1 to T4 respectively; the absolute values shift by about a third across that range, but the ranking of the treatments, the separation of the control from the supplemented treatments and the significance of the treatment effect (*p* = 0.007) are preserved at every value tested. The conclusion that supplementation improved the efficiency of microbial nitrogen supply is therefore robust to the assumption, even though the absolute efficiency reported should be read as an estimate conditional on it.

A comment is needed on the combination arm, because it is the feature of the design most open to over-interpretation. T4 supplied 5 g of each additive and produced digestibility, fermentation and microbial responses that could not be separated statistically from either additive alone at 10 g. That observation is not evidence of equivalence, additivity or substitution, and the present experiment cannot test any of them: the treatment structure is not factorial, because no single-additive arms at 5 g were included, so no interaction term exists, and with six residual degrees of freedom the power to detect a moderate difference between T4 and the single additives is low. The result is therefore offered as a hypothesis that the two additives may converge on a common limiting step in this diet, and not as a conclusion. The only comparable factorial in ruminants, conducted in goats, likewise found that yeast and dried kratom leaf each improved digestibility, reduced protozoa and estimated methane and increased propionate, with no interaction detected for intake or for bacterial and fungal populations [17], while combination effects of a live yeast with a bioactive plant or algal source have elsewhere depended strongly on host species and substrate [38]. A full factorial including single-additive treatments at 5 g, with sufficient replication for an equivalence test to be meaningful, is the necessary next step before any dose-sparing recommendation could be made.

Several limitations should be borne in mind, and they bear directly on how far the findings can be carried. The experiment used four young Thai native heifers of about 140 kg offered a single basal ration over four 21-day periods, and it measured digestion and ruminal function rather than production. Improved nutrient digestibility is a necessary but not a sufficient condition for better growth or feed conversion, and the design cannot speak to liveweight gain, carcass outcomes or feed efficiency in a commercial finishing system; extrapolation to older or heavier cattle, to other basal rations, or to longer feeding periods is not warranted on this evidence. The 4 × 4 Latin square leaves six residual degrees of freedom, so the power to detect moderate differences is low and a non-significant result should not be read as evidence of no effect. Only the condensed tannin concentration of the leaf powder was determined, so the alkaloid, saponin and total phenolic contributions of the batch fed can be inferred from the literature but not quantified, and only one full dose and one half dose of MSP were tested, so it is not known whether the responses observed lie on the ascending, plateau or descending part of the dose–response curve. Rumen microbial populations were enumerated by direct microscopy, which quantifies total counts but cannot resolve shifts among specific functional taxa such as cellulolytic bacteria or methanogens; inferences about altered functional communities therefore remain speculative without molecular profiling. Ruminal pH, ammonia nitrogen and volatile fatty acids were measured at a single time point, 4 h after the morning feeding, so neither the diurnal pattern of ruminal conditions nor any treatment by time interaction could be examined, and the values reported describe the rumen at that point in the feeding cycle rather than a daily mean; urine was likewise sampled as a single spot sample per animal and period rather than by total collection, so the purine derivative to creatinine approach used here rests on one point of the diurnal excretion curve and on an assumed creatinine excretion rate rather than on a measured daily output, which is a recognised limitation of the method and the reason the absolute purine derivative values are reported as estimates. Neutral detergent fiber was assayed without heat-stable α-amylase, which in a ration containing 16% cassava chip may overestimate the fiber fraction through residual starch.

A separate consideration applies to the safety of feeding kratom to food-producing animals. *M. speciosa* leaf contains the indole alkaloids mitragynine and 7-hydroxymitragynine, which are agonists at opioid receptors and are pharmacologically active in humans at low doses. No residue data exist for mitragynine or its metabolites in the meat or milk of ruminants, no withdrawal period has been established, and no maximum residue limit has been set in Thailand or elsewhere. Throughout the experiment the animals were observed daily by trained staff for feed refusal, demeanour, faecal consistency and any clinical abnormality; no abnormal clinical signs were recorded in any animal in any period. The available animal data address tolerance rather than residues: feeding dried kratom leaf to goats for 90 days at up to 6.66 g/day produced no adverse effects on growth performance, haematology or carcass traits [13], and the cattle in the present study showed no adverse response over four 21-day periods. A trial of this length, with intake unchanged, is evidence of short-term tolerance only and cannot be read as evidence of long-term safety. Tolerance in the animal, however, says nothing about deposition in edible tissue, and the two questions should not be conflated. Although kratom was decriminalized in Thailand in 2021 and the leaf is now traded openly as an agricultural commodity, its use in feed for food-producing animals is not covered by any specific regulatory framework. The present results therefore support further investigation of kratom leaf as a feed additive, but do not support its commercial adoption in beef production until tissue residue studies and a formal withdrawal assessment have been completed.

## 5. Conclusions

Supplementation of Thai native beef cattle with *M. speciosa* leaf powder at 10 g/head/day, *S. cerevisiae* at 10 g/head/day, or their combination at 5 g/head/day of each, improved the apparent total-tract digestibility of dry matter, organic matter, crude protein and acid detergent fiber, with a tendency towards higher neutral detergent fiber digestibility, and did so without depressing feed intake. Supplementation raised ruminal pH, significantly in the two treatments containing yeast, reduced ruminal ammonia nitrogen and blood urea nitrogen, increased bacterial populations while suppressing protozoa, shifted fermentation towards propionate, reduced estimated methane production, and increased microbial nitrogen supply by 38.7–45.7%. The efficiency with which dietary nitrogen was converted into microbial protein rose by 25.0–30.0% (*p* = 0.007). The combination at half the dose of each additive produced responses that could not be separated statistically from either additive alone at the full dose. Because the design was not factorial and the residual degrees of freedom were few, this is reported as a hypothesis for testing rather than as evidence of equivalence or of a dose-sparing effect. Longer-term studies incorporating growth performance, direct methane measurement, molecular characterization of the rumen microbiome and tissue residue analysis are recommended.

## Figures and Tables

**Table 3:** Effect of *Mitragyna speciosa* leaf powder and *Saccharomyces cerevisiae* supplementation on apparent total-tract nutrient digestibility in Thai native beef cattle.

Item	T1	T2	T3	T4	SEM	*p*-Value
Nutrient digestibility, %						
Dry matter	60.40 ^b^	64.77 ^ab^	65.00 ^ab^	65.40 ^a^	1.02	0.040
Organic matter	62.60 ^b^	66.72 ^ab^	67.40 ^a^	65.40 ^ab^	0.89	0.034
Crude protein	56.85 ^b^	64.69 ^a^	65.00 ^a^	63.77 ^a^	0.36	<0.001
Neutral detergent fiber	55.48	59.63	60.13	59.17	0.98	0.052
Acid detergent fiber	31.57 ^b^	41.12 ^a^	40.25 ^a^	40.45 ^a^	1.07	0.002

^a, b^: Means within a row with different superscripts differ (*p* < 0.05). Treatment codes are as in Table 2. Orthogonal contrast *p*-values (control vs. supplemented; MSP vs. SC; combination vs. the two single additives): dry matter 0.007, 0.880, 0.694; organic matter 0.009, 0.606, 0.176; crude protein < 0.001, 0.560, 0.049; neutral detergent fiber 0.010, 0.730, 0.578; acid detergent fiber < 0.001, 0.585, 0.863.

**Table 4:** Effect of *Mitragyna speciosa* leaf powder and *Saccharomyces cerevisiae* supplementation on rumen ecology and blood urea nitrogen in Thai native beef cattle.

Item	T1	T2	T3	T4	SEM	*p*-Value
Ruminal pH	6.48 ^b^	6.60 ^b^	6.80 ^a^	6.83 ^a^	0.035	0.001
Ammonia nitrogen (NH_3_-N), mg/dL	16.65 ^a^	12.78 ^b^	12.08 ^b^	13.10 ^b^	0.287	<0.001
Blood urea nitrogen (BUN), mg/dL	14.50 ^a^	11.75 ^b^	12.90 ^ab^	12.25 ^b^	0.456	0.023

^a, b^ Means within a row with different superscripts differ (*p* < 0.05). Treatment codes are as in Table 2. Orthogonal contrast *p*-values (control vs. supplemented; MSP vs. SC; combination vs. the two single additives): ruminal pH < 0.001, 0.007, 0.028; ammonia nitrogen < 0.001, 0.135, 0.103; blood urea nitrogen 0.006, 0.125, 0.898.

**Table 5:** Effect of *Mitragyna speciosa* leaf powder and *Saccharomyces cerevisiae* supplementation on ruminal volatile fatty acid profile and estimated methane production in Thai native beef cattle.

Item	T1	T2	T3	T4	SEM	*p*-Value
Total volatile fatty acids, mmol/L	102.33	102.95	102.63	103.15	1.148	0.957
Volatile fatty acid profile, mol/100 mol						
Acetate (C_2_)	66.40	65.65	65.45	64.40	0.450	0.097
Propionate (C3)	19.88 ^b^	22.28 ^a^	22.18 ^a^	21.93 ^a^	0.250	0.001
Butyrate (C4)	13.73	12.08	12.38	13.68	0.570	0.180
Acetate to propionate ratio	3.34 ^a^	2.95 ^b^	2.96 ^b^	2.94 ^b^	0.036	<0.001
Estimated CH_4_ ^1^ mol/100 mol	29.90 ^a^	28.25 ^b^	28.30 ^b^	28.42 ^b^	0.164	0.001

^1^ Estimated stoichiometrically from the molar volatile fatty acid profile according to Moss et al. [25]: CH_4_ = 0.45 (acetate) − 0.275 (propionate) + 0.40 (butyrate). Methane was not measured directly. ^a, b^: Means within a row with different superscripts differ (*p* < 0.05). Treatment codes are as in Table 2. Orthogonal contrast *p*-values (control vs. supplemented; MSP vs. SC; combination vs. the two single additives): total volatile fatty acids 0.675, 0.848, 0.805; acetate 0.055, 0.764, 0.082; propionate < 0.001, 0.786, 0.364; butyrate 0.174, 0.723, 0.083; acetate to propionate ratio < 0.001, 0.872, 0.736; estimated methane < 0.001, 0.812, 0.497.

**Table 6:** Effect of *Mitragyna speciosa* leaf powder and *Saccharomyces cerevisiae* supplementation on rumen microbial populations in Thai native beef cattle.

Item	T1	T2	T3	T4	SEM	*p*-Value
Total bacteria: ×10^9^ cells/mL	2.40 ^b^	4.45 ^a^	3.60 ^ab^	3.90 ^a^	0.272	0.009
Protozoa, ×10^6^ cells/mL	5.60 ^a^	2.40 ^b^	2.70 ^b^	2.20 ^b^	0.385	0.002
Fungal zoospores, ×10^4^ cells/mL	2.03	2.15	1.78	2.15	0.298	0.790

^a, b^: Means within a row with different superscripts differ (*p* < 0.05). Treatment codes are as in Table 2. Orthogonal contrast *p*-values (control vs. supplemented; MSP vs. SC; combination vs. the two single additives): total bacteria 0.002, 0.069, 0.720; protozoa < 0.001, 0.602, 0.486; fungal zoospores 1.000, 0.408, 0.626.

**Table 7:** Effect of *Mitragyna speciosa* leaf powder and *Saccharomyces cerevisiae* supplementation on purine derivative excretion and microbial protein synthesis in Thai native beef cattle.

Item	T1	T2	T3	T4	SEM	*p*-Value
Purine derivatives, mmol/d						
Total purine derivatives excreted	44.58 ^b^	55.96 ^a^	57.69 ^a^	55.63 ^a^	1.40	0.002
Purine derivatives absorbed ^1^	33.92 ^b^	47.31 ^a^	49.43 ^a^	47.05 ^a^	1.65	0.002
Microbial nitrogen supply ^2^, g N/d	24.66 ^b^	34.40 ^a^	35.93 ^a^	34.21 ^a^	1.20	0.002
Organic matter digested in the rumen (DOMR), kg/d	1.60 ^b^	1.78 ^a^	1.79 ^a^	1.73 ^ab^	0.027	0.008
Efficiency of microbial N supply (EMNS) ^3^ g N/kg OMDR	15.44 ^b^	19.30 ^a^	20.07 ^a^	19.91 ^a^	0.65	0.007

^1^ Purine derivatives absorbed, calculated from urinary excretion according to Chen and Gomes [26]. ^2^ Microbial nitrogen supply = 70X/(0.83 × 0.116 × 1000), where X is absorbed purine derivatives (mmol/d) [26,31]; the equivalent microbial crude protein supply is obtained as microbial nitrogen × 6.25. ^3^ EMNS = microbial nitrogen supply (g N/d)/DOMR (kg/d), where DOMR is the organic matter apparently digested in the rumen, calculated for each animal within each period as organic matter intake × total-tract organic matter digestibility × 0.65, on the assumption that ruminal digestion accounted for 65% of total-tract organic matter digestion. ^a, b^ Means within a row with different superscripts differ (*p* < 0.05). Orthogonal contrast *p*-values (control vs. supplemented; MSP vs. SC; combination vs. the two single additives): total purine derivatives excreted < 0.001, 0.414, 0.513; purine derivatives absorbed < 0.001, 0.402, 0.539; microbial nitrogen supply < 0.001, 0.402, 0.539; DOMR 0.002, 0.869, 0.127; EMNS 0.001, 0.438, 0.784.

## Data Availability

The data presented in this study are available on request from the corresponding author.
